# The Effect of Acupuncture on Brain Iron Deposition and Body Iron Metabolism in Vascular Cognitive Impairment: Protocol for a Randomized Controlled Trial

**DOI:** 10.2196/56484

**Published:** 2024-06-17

**Authors:** Mingli Wu, Lulu Chen, Yamin Wang, Yunpeng Li, Yuqi An, Ruonan Wu, Yuhan Zhang, Jing Gao, Kaiqi Su, Xiaodong Feng

**Affiliations:** 1 Rehabilitation Center The First Affiliated Hospital of Henan University of Chinese Medicine Zhengzhou China; 2 First Clinical Medical College Henan University of Chinese Medicine Zhengzhou China; 3 Medical College of Rehabilitation Henan University of Chinese Medicine Zhengzhou China

**Keywords:** acupuncture, vascular cognitive impairment, iron metabolism, mechanisms explored, clinical trial, needling technique, dry needling, acupunctures, activities of daily living, iron, prevalence, cerebrovascular diseases, vascular dementia, vascular, traditional Chinese method, Chinese methods

## Abstract

**Background:**

Vascular cognitive impairment (VCI) persistently impairs cognition and the ability to perform activities of daily living, seriously compromising patients’ quality of life. Previous studies have reported that disorders of serum iron metabolism and iron deposition in the brain can lead to inflammation, abnormal protein aggregation and degeneration, and massive neuronal apoptosis in the central nervous system, which in turn leads to a progressive decline in cognitive processes. Our previous clinical studies have found acupuncture to be a safe and effective intervention for treating VCI, but the specific mechanisms require further exploration.

**Objective:**

The objective of the trial is to evaluate the clinical efficacy of Tongdu Xingshen acupuncture and to investigate whether it can improve VCI by regulating brain iron deposition and body iron metabolism.

**Methods:**

In total, 42 patients with VCI and 21 healthy individuals will participate in this clinical trial. The 42 patients with VCI will be randomized into acupuncture and control groups, while the 21 healthy individuals will be in the healthy control group. Both the control and acupuncture groups will receive conventional medical treatment and cognitive rehabilitation training. In addition, the acupuncture group will receive electroacupuncture treatment with Tongdu Xingshen for 30 minutes each time, 6 times a week for 4 weeks. Meanwhile, the healthy control group will not receive any intervention. All 3 groups will undergo baseline assessments of brain iron deposition, serum iron metabolism, and neuropsychological tests after enrollment. The acupuncture and control groups will be evaluated again at the end of 4 weeks of treatment, as described earlier. By comparing neuropsychological test scores between groups, we will examine the efficacy of Tongdu Xingshen acupuncture in treating VCI. Additionally, we will test the correlations between neuropsychological test scores, brain iron deposition, and body iron metabolism indexes to explore the possible mechanisms of Tongdu Xingshen acupuncture in treating VCI.

**Results:**

Participants are currently being recruited. The first participant was enrolled in June 2023, which marked the official start of the experiment. As of the submission of the paper, there were 23 participants. The recruitment process is expected to continue until June 2025, at which point the processing and analysis of data will begin. As of May 15, 2024, up to 30 people have been enrolled in this clinical trial.

**Conclusions:**

This study will provide data on the effects of Tongdu Xingshen acupuncture on cerebral iron deposition as well as somatic iron metabolism in patients with VCI. These results will help to prove whether Tongdu Xingshen acupuncture can improve VCI by regulating brain iron deposition and body iron metabolism, which will provide the clinical and theoretical basis for the wide application of acupuncture therapy in VCI rehabilitation.

**Trial Registration:**

China Clinical Registration Agency ChiCTR2300072188; https://tinyurl.com/5fcydtkv

**International Registered Report Identifier (IRRID):**

PRR1-10.2196/56484

## Introduction

Vascular cognitive impairment (VCI) is a series of clinical syndromes that encompasses mild cognitive impairment to severe vascular dementia (VD) caused by cerebrovascular disease and its risk factors [1]. VCI accounts for 15% to 20% of all patients with dementia in North America and Europe, and up to 30% of patients with dementia in Asia, making it the second most prevalent type of dementia after Alzheimer disease [2]. The persistent and progressive decline of higher brain functions, such as learning, memory, and executive functions, caused by VCI is a difficult problem in clinical rehabilitation, which seriously affects patients’ quality of life and involves a severe burden for families and society. Although donepezil and memantine may slow the progression of VCI, there are currently no effective clinical drug options for VCI from a clinical perspective [3,4]. Although nonpharmacological treatments such as aerobic exercise and cognitive rehabilitation training, as well as early control of risk factors such as blood pressure, lipids, and blood glucose, have been reported to have positive effects on the prevention and treatment of VCI, a range of issues limit the effective treatment of VCI, including a lack of clinical research, low compliance, lengthy interventions, and variable efficacy [5-8]. Therefore, it is important to find safe, effective, and widely applicable treatment methods for the clinical treatment and development of VCI.

Numerous studies have indicated that traditional Chinese medicine (TCM) can play an important role in the treatment of VCI [9,10]. In particular, acupuncture has the unique advantages of simplicity, convenience, safety, and effectiveness in the treatment of VCI [11]. According to the understanding of VCI in Chinese medicine, in which the “brain” and “governor vessel” play key roles in regulating the “vital spirit” and brain function, the “deficiency of the medulla oblongata and loss of the use of the spiritual mechanism” is considered to be the fundamental pathogenesis of VCI, leading to the development of the Tongdu Xingshen treatment principle. Several prior clinical studies in our laboratory and meta-analyses published by other researchers have demonstrated that acupuncture is effective in improving cognitive function and enhancing overall outcomes in patients with a history of stroke [12-14]. However, the in-depth mechanisms of Tongdu Xingshen acupuncture treatment for VCI require further validation and exploration.

The pathogenesis of VCI is complex. Previous studies of the pathogenesis of VCI have mainly focused on blood-brain barrier disruption, secondary inflammation, excitatory amino acid toxicity, cell autophagy, and apoptosis [15,16]. With the discovery of ferroptosis and the in-depth study of its mechanism of occurrence, more and more scholars believe that it plays an important role in central nervous system diseases [17,18]. By exploring the relationship between serum iron metabolism, brain iron deposition, and cognitive impairment, researchers in various countries, including China, have found that iron deposition can lead to the development of inflammation, abnormal protein aggregation, and degeneration in the central nervous system, which in turn causes a progressive decline in cognitive function [19,20].

Quantitative susceptibility mapping (QSM) is a postprocessing technique developed in recent years on the basis of sensitivity-weighted imaging (SWI), which enables to quantify the magnetic susceptibility properties of tissues in vivo [21,22]. The ability to measure brain iron content, which is quantified by the magnetization rate values obtained from QSM calculations, raises the possibility of investigating ferroptosis in VCI.

Therefore, the proposed study will examine brain iron deposition and body iron metabolism as entry points and use a clinical randomized controlled study design to investigate the clinical efficacy and possible mechanism of action of Tongdu Xingshen acupuncture to treat VCI with a view to providing a scientific basis for the clinical application of acupuncture.

## Methods

### Research Objectives

The primary objectives of this randomized controlled trial will be twofold: (1) to observe the clinical efficacy of Tongdu Xingshen acupuncture in patients with VCI and provide feasible and affordable acupuncture treatment for patients with VCI and (2) to investigate whether Tongdu Xingshen acupuncture improves the clinical performance of patients with VCI by affecting brain iron deposition and body iron metabolism and reveal the potential central and peripheral mechanisms of acupuncture in the treatment of vascular cognitive disorder.

### Trial Design and Setting

**Figure 1:**
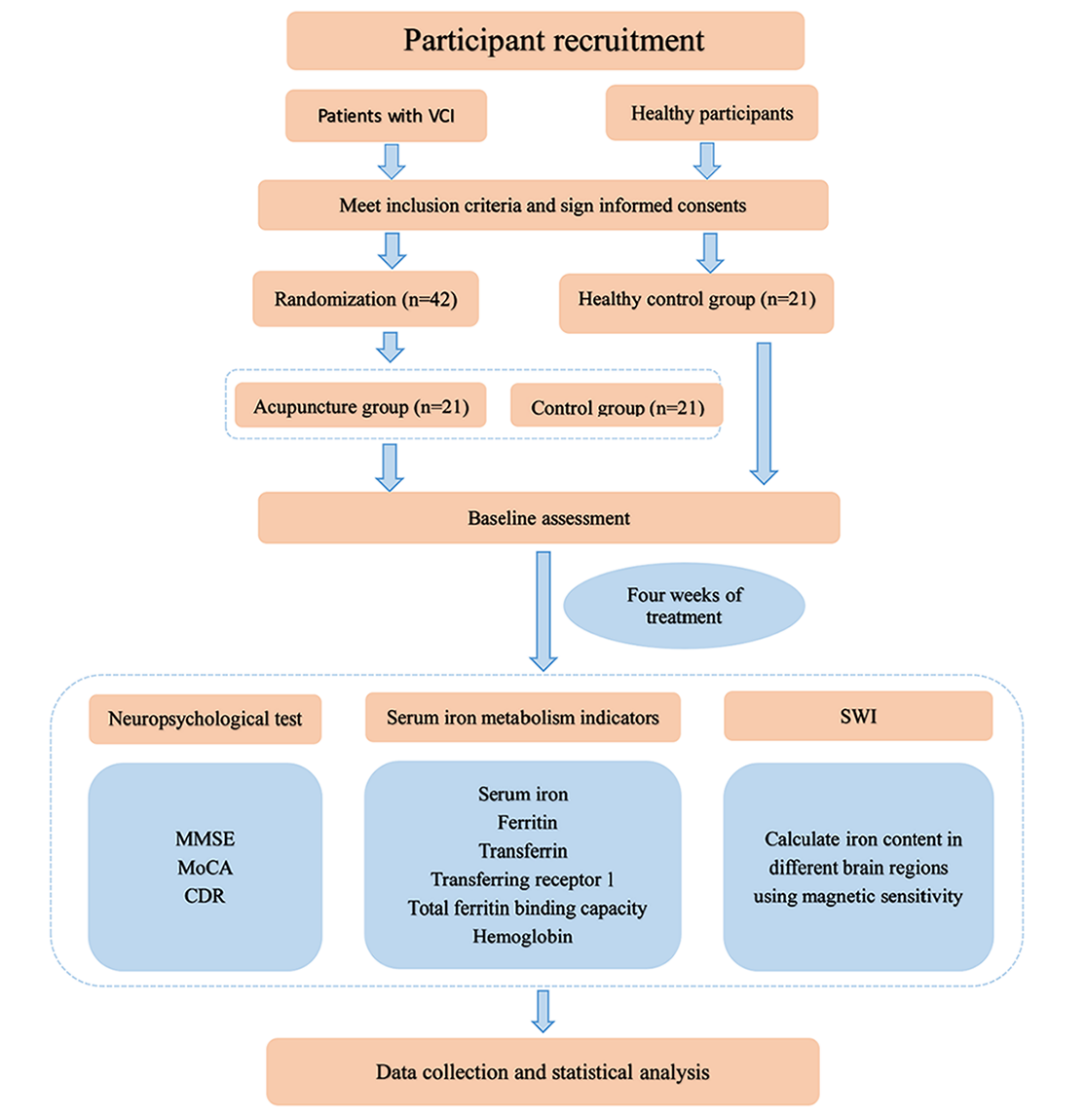
Flowchart of the trial. CDR: Clinical Dementia Rating; MMSE: Mini-Mental State Examination; MoCA: Montreal Cognitive Assessment; SWI: sensitivity-weighted imaging; VCI: vascular cognitive impairment.

The proposed protocol involves a randomized controlled clinical trial to investigate whether Tongdu Xingshen acupuncture can improve the clinical performance of patients with VCI by regulating brain iron deposition and body iron metabolism. In total, 42 patients with VCI hospitalized at the First Affiliated Hospital of Henan University of Traditional Chinese Medicine from May 2023 to December 2025 and 21 healthy participants will be included in the study. Participants will be divided into acupuncture, conventional treatment, and healthy control groups according to a ratio of 1:1:1, with 21 participants in each group. The control group (n=21) will receive conventional medical and cognitive rehabilitation treatment, the acupuncture group (n=21) will receive professional acupuncture treatment by a professional acupuncturist on the basis of the conventional treatment, and the healthy control group (n=21) will not receive any intervention. All of the above treatments will be performed for 4 weeks, 6 times per week. Brain iron deposition and serum iron metabolism will be measured at week 0 (baseline) and week 4 of treatment using SWI and blood biochemistry. In parallel, a neuropsychological test battery including the Mini-Mental State Examination (MMSE), Montreal Cognitive Assessment (MoCA), and Clinical Dementia Rating will be administered to evaluate comprehensive cognitive function and clinical symptoms. Details of the proposed study process are presented in Figure 1 and Table 1.

**Table 1:** Timing of treatment assessments and data collection.

Time point			Enrollment (week –1)		Baseline assessment (week 0)		Treatment phase (weeks 1-4)		Assessment of efficacy (week 5)
Sign informed consent			✓						
Demographic information			✓						
Medical history			✓						
Disease history			✓						
Randomization			✓						
**Interventions**									
	Acupuncture plus routine treatment			✓		✓			
	Routine treatment			✓		✓			
	Assessments			✓					
**Cognitive function assessments**									
	MMSE^a^			✓				✓	
	MoCA^b^			✓				✓	
	CDR^c^			✓				✓	
**Blood detection**									
	Serum iron metabolism indicators			✓				✓	
	SWI^d^ scanning								
	Brain iron content			✓				✓	
Safety evaluation					✓		✓		
Adverse event					✓		✓		

^a^MMSE: Mini-Mental State Examination.

^b^MoCA: Montreal Cognitive Assessment.

^c^CDR: Clinical Dementia Rating.

^d^SWI: sensitivity-weighted imaging.

### Participants and Recruitment Strategy

Participants in this study will be recruited mainly through the inpatient system of the First Affiliated Hospital of Henan University of Traditional Chinese Medicine, and we will also use the hospital’s website and WeChat public number platform for promotion and recruitment. Patients with VCI and healthy participants who are interested and whose initial screening meets our recruitment needs will be fully informed of the entire study procedure, benefits, and risks. We will conduct this recruitment with full respect for each participant’s individual wishes. We also guarantee that the right of each participant to withdraw from the study will be respected at all stages of the clinical trial.

### Inclusion and Exclusion Criteria

#### Inclusion Criteria

Inclusion criteria are as follows: (1) aged 18-75 years, male or female; (2) patient or informant reports of memory loss or other cognitive impairment lasting for at least 3 months and not using any medication to improve cognitive function prior to enrollment; (3) diagnostic criteria for VCI are met (Western medical diagnostic criteria refer to the *2019 Chinese Guidelines for the Diagnosis and Treatment of Vascular Cognitive Disorder* and Chinese medical diagnostic criteria refer to the *Diagnostic, Identification and Efficacy Determination Criteria for Vascular Dementia*); (4) imaging evidence (computed tomography or magnetic resonance imaging) to support cerebrovascular disease; (5) clear cognitive dysfunction, as assessed by the MMSE (no education: MMSE score≤17, primary school: MMSE score≤20, or junior school or above: MMSE score≤24) and MoCA (MoCA score < 26 points); and (6) consciousness and stable vital signs.

#### Exclusion Criteria

Exclusion criteria are as follows: (1) occurrence of other brain diseases that affect cognitive function, such as intracranial occupying lesions, traumatic brain injury, arteriovenous aneurysms, and intracranial infections; (2) cognitive impairment caused by hemorrhagic stroke; (3) diseases that seriously affect the cognitive examination such as abnormal visual and auditory functions, and psychiatric abnormalities; (4) history of significant cognitive decline or dementia prior to current onset; (5) other serious diseases such as immune diseases, endocrine diseases, abnormal liver, and kidney function; (6) pregnant and lactating women; and (7) coagulation disorders and treatment site infections.

### Rejection Criteria

Rejection criteria are as follows: (1) individuals who were mistakenly included without a diagnosis of VCI; (2) individuals who did not complete the treatment cycle in the enrolled study cases; (3) serious adverse events or complications that make it inappropriate to continue the experiment; (4) participants spontaneously shed, miss visits, or die during observation; and (5) receiving medications and treatments that have a significant effect on outcomes during the trial.

### Sample Size

Previous clinical studies on patients with VCI treated with acupuncture reported that the scores on the MoCA scale of acupuncture-treated and base-treated patients with VCI at 4 weeks were mean 26.13 (SD 5.33) and mean 22.66 (SD 5.38), respectively. We set the significance level at .05 and calculated the sample size as follows:

N = 2 [(*Z*_α/2_ + *Z*_β_) σ / δ]^2^

N is the sample size in each group, and σ and δ represent the larger SD and mean difference between the 2 groups with values of 5.38 and 3.47, respectively. When α=.05 and β=.1, checking the table shows that *Z*_α/2_=1.96 and *Z*_β_=1.282. Substituting the above data into the formula, a minimum of 64 patients in the randomized acupuncture group or control group will be required, considering a 20% shedding rate.

A review of relevant studies indicates that in magnetic resonance imaging–based studies of cognitive impairment, statistical significance can be achieved with 12 or more cases [23,24]. Because of limitations in the experimental conditions (high scanning cost of magnetic resonance imaging and the need for high levels of participant cooperation during scanning), we estimate that approximately one-third of the estimated sample of patients with VCI will be included as study participants, and finally, 42 patients with VCI and 21 healthy controls will be included.

### Randomization

To ensure balanced comparability between groups, we will use SPSS software (version 25.0; IBM Corp) to generate a random sequence, and 42 participants with VCI who meet the inclusion criteria will be randomly assigned to the acupuncture and control groups at a ratio of 1:1. In addition, the 42 numbered random sequences as well as the grouping scheme will be placed independently in sealed opaque envelopes, which will be opened only when a patient with VCI is enrolled. The randomization and concealment processes described earlier will be carried out by an independent third-party statistician who is not involved in any subsequent parts of this study.

### Blinding

Because of the limitations of the protocol and the specific nature of acupuncture treatment, it will not be possible to blind the participants and the acupuncturists. However, to improve the quality and reliability of this study, the researchers will be blinded during the efficacy assessment, data collection and management, and statistical analysis of the results. Thus, the efficacy assessors, data collection and management personnel, and outcome analysis personnel will not be aware of the grouping of the trial and will not be allowed to communicate with each other or with the participants about the treatment. In addition, to prevent bias in the results by nonblinded participants or researchers, this study will only involve individuals who do not have a conflict of interest or preconceived position.

### Intervention

#### Overview

Different groups will receive different interventions: the acupuncture group will receive acupuncture in addition to conventional medical treatment and cognitive rehabilitation training, the control group will receive conventional medical treatment and cognitive rehabilitation training only, and healthy participants will not receive any treatment. All of the above treatments will be performed by physicians and therapists who are certified and have more than 5 years of clinical experience. Below we describe the specific interventions for the 2 intervention groups.

#### Acupuncture Group

On the basis of conventional medical treatment and cognitive rehabilitation training, we will use Shenting and Baihui for electroacupuncture treatment. The specific acupuncture point positioning standards refer to *Acupuncture and Moxibustion* (“14th Five-Year Plan” National Higher Education Planning Textbook for the Chinese Medicine Industry). The specific method is as follows: the patient is placed in a supine position, and after routine disinfection of the Shenting and Baihui points with 75% alcohol cotton balls, the patient is stabbed backward with Hua Tuo brand (0.25 mm×25 mm) acupuncture needles in a flat direction (needle body and skin surface layer 15 degrees) of Baihui 0.8 to 1.0 inch and Shenting 0.5 inch. The electroacupuncture therapy instrument (SDZ-II; Jiangsu Medical Supplies Co) is connected to acupuncture needles after the patient gets qi (local soreness), and the waveform is continuous for the first 15 minutes, at an intensity that is tolerated by the patient, and then changes to a sparse waveform after 15 minutes to prevent electrical adaptation. After 30 minutes of electroacupuncture, the electroacupuncture instrument is removed; the needles are not removed and are retained for 1 hour. Cognitive function training is performed during the retention period, and the needles are twisted once at the beginning and once at 30 minutes, until the end of cognitive function training. The acupuncture needles are then removed. The electroacupuncture treatment will be performed once a day, 6 times a week, for 4 weeks.

#### Control Group

The control group will receive only conventional medical treatment as well as cognitive rehabilitation training. Specific treatment will include symptomatic treatment for the patient’s underlying diseases, such as controlling blood pressure, blood sugar, and blood lipids, antiplatelet aggregation, and nourishing brain cells. At the same time, according to the patient’s condition, a combination of manual training and computer training will be used to perform conventional cognitive function training such as category classification, rule (response) inhibition, plan analysis, reasoning and comprehension, working memory, and synthesis ability, for 1 hour per session, 6 times a week, for 4 weeks.

#### Healthy Control Group

The healthy control group will receive no intervention.

### Outcome Measurements

#### Overview

The 3 main areas of assessment will include neuropsychological tests, SWI, and serum iron metabolism. Researchers who are blinded to the treatment allocation will carry out all treatment evaluations.

#### Neuropsychological Tests

Cognitive function will be assessed at baseline and after 4 weeks of treatment by a professional evaluator using neuropsychological tests, and the evaluator will not be aware of the current grouping. The following are the neuropsychological test scales that will be used in this experiment.

#### MMSE Scale

The MMSE scale consists of 30 questions (1 point per question) in 7 areas: temporal orientation, place orientation, immediate memory, attention and calculation, delayed memory, language, and visuospatial ability [25]. Scores on this scale are closely related to literacy level, and it is generally accepted that people with illiteracy (≤17), primary school education (≤20), and junior high school and above education (≤24) have different degrees of cognitive dysfunction. Because of its simplicity, time-saving features, convenience of administration, and wide range of applications, the MMSE scale is used for the initial screening of different types of cognitive impairment and dementia.

#### MoCA Scale

The MoCA scale was developed by Tsai et al [26] in 2004 for rapid screening of mild cognitive impairment and has better sensitivity than the MMSE. The MoCA scale assesses cognitive domains including attention and concentration, executive function, memory, language, visual-structural skills, abstract thinking, numeracy, and orientation, with a total scale score of 30, and test results showing a normal value of ≥26.

#### Clinical Dementia Rating

In Clinical Dementia Rating, the physician obtains information by talking to the patient and family to quickly complete an assessment of the patient’s level of cognitive impairment [27]. The scale’s assessment domains include memory, orientation, judgment and problem-solving, work and social interaction, family life and personal hobbies, and independent living skills. Each of the above 6 functions was evaluated at 5 levels, from no impairment to severe impairment. The 6 abilities were combined into a total score that is based on the overall scoring criteria, with the results expressed as 0, 0.5, 1, 2, or 3, which are considered to represent normal, suspicious, mild, moderate, and severe dementia, respectively.

#### Serum Iron Metabolism–Related Indicators

A total of 4 mL of blood will be collected from patients in the morning on an empty stomach. The supernatant will then be extracted after centrifugation at 3000 revolution/min for 15 minutes at 4 °C. The levels of iron, ferritin, transferrin, transferrin receptor 1, total iron binding capacity, and hemoglobin will be measured using enzyme-linked immunosorbent assay.

#### High-Resolution Magnetically SWI

All participants will undergo SWI, and the data will be postprocessed and analyzed using Susceptibility Mapping and Phase Artifacts Removal Toolbox software (The Math Works Inc) to obtain QSM images. Signal processing in Nuclear Magnetic Resonance software (Philips Healthcare Netherlands Ltd) will then be used to detect and quantitate the corrected phase value in the region of interest, including bilateral parietal cortex, frontal white matter, caudate nucleus, putamen, globus pallidus, thalamus, red nucleus, substantia nigra, hippocampus, and dentate nucleus [21,22,28]. Care will be taken to avoid blood vessels, calcifications, and visible lesions during the measurements. Each site will be measured twice, and the average of the 2 measurements will be taken. The magnitude of magnetization will be used to indicate the level of iron content of the tissue.

### Safety Evaluation and Adverse Events

We will minimize the occurrence of adverse events in this clinical trial to ensure the safety of each participant. A comprehensive physical safety examination will be performed on all volunteers before and after treatment, including vital signs (heart rate, blood pressure, and respiration), routine blood and urine tests, biochemical function tests, electrocardiography, and liver and kidney function tests. Possible adverse reactions in this study include pain during acupuncture, fainting, and skin breakage or other injury caused by improper handling. If the patient experiences acupuncture fainting because of weakness or excessive stress, the acupuncture treatment will be stopped immediately, and the patient will be placed in a comfortable position and given warm water until they recover. In addition, if pain or systemic adverse reactions are caused by excessive current and voltage intensity, the electroacupuncture instrument will be turned off immediately. After confirming that the patient has no adverse reactions, the electroacupuncture instrument will be properly connected and used correctly according to the operational requirements. We will not only record the occurrence of any of the abovementioned adverse events in the case report book but will also actively deal with them.

### Data Collection and Management

All participants’ information will be recorded in the case report form in a true, complete, accurate, and timely manner, and data entry will be performed using the Clinical Monitor Data Manager (Microsoft Corp) after completion of at least 5 case report forms. We will use a data entry program that is prepared by computer software for the double entry of data, which keeps the participant’s personal information strictly confidential during the entry process. To prevent data leakage and loss, these data will be stored by our data researchers on servers with passwords and access restrictions and will not be shared with third parties outside of the data recorders and data administrators without the express permission of the research team leader. The quality control team will regularly review the accuracy and reliability of our data, and if there is no objection to these data, we will lock the data. No further changes will be allowed to the locked data file, and the database will be handed over to the statistical analysts for statistical analysis as required by the statistical plan. All of the above data collectors and managers will be involved in this clinical trial only after passing a standardized training and assessment, and more importantly, an agreement will be signed with the data collectors and managers to ensure that they will not disclose any personal information of the participants.

### Statistical Analysis

All data will be statistically analyzed by professional statisticians using SPSS software (version 25.0). If the data involved satisfy the characteristic properties of a normal distribution with uniform variance, we will use the mean and SD to describe them; conversely, if the data are not normally distributed, we will use the median and IQR to describe them. All statistical results will be considered statistically significant at *P*<.05.

Neuropsychological scale scores, iron metabolism–related indicators, and brain iron deposition are all measurement data. Normally distributed data with uniform variance among 3 groups will be analyzed using 1-way ANOVA, followed by multiple comparisons using the least significant difference test, whereas those with nonnormal distributions will be compared using the Kruskal-Wallis test followed by the Mann-Whitney *U* test with Bonferroni correction. Correlations between neuropsychological scale scores and serum iron metabolism indexes and brain iron deposition in patients with VCI will be assessed using Pearson correlation analysis.

### Quality Control

To ensure the quality of the proposed experiment, we will conduct unified and standardized training for the researchers in each role before the start of this study. Researchers will then be tested, and only competent researchers will participate in this study. Additionally, to improve participant compliance, we will conduct regular health education activities, enhance humane care, and develop a long-term follow-up plan. Finally, with reference to the requirements in the Specifications for Quality Control and Quality Assurance of Clinical Research in Chinese Medicine, a 4-level quality monitoring system will be established to monitor the clinically collected data on a regular monthly basis to ensure the authenticity and reliability of the study results.

### Ethical Considerations

This study will adhere to the principles outlined in the Declaration of Helsinki, ensuring that no additional harm or risks are imposed on the participants. Ethics approval has already been granted by the ethics committee of the First Affiliated Hospital of Henan University of Traditional Chinese Medicine on May 6, 2023 (ethics reference 2023HL-141-01). Prior to the start of the clinical trial, all participants will be fully informed of the purpose of the trial, the procedures involved, the expected duration, and the potential risks and benefits and will voluntarily provide written informed consent. To protect the privacy of participants, their real names will not appear in the case reports, and they have the right to opt out of any section of the experiment. In addition, all participants will receive a Chinese medicine gift pack valued at US $30.

## Results

Participants are currently being recruited. The first participant was enrolled in June 2023, which marked the official start of the experiment. As of the submission of the paper, there were 23 participants. The recruitment process is expected to continue until June 2025, at which point the processing and analysis of data will begin. As of May 15, 2024, up to 30 people have been enrolled in this clinical trial.

## Discussion

### Overview

With the aging of society and the increasing prevalence of cerebrovascular diseases, the number of patients with VCI is increasing. If VCI is not effectively controlled at an early stage, patients may exhibit further deterioration and eventually develop VD [1]. More seriously, there is currently no effective conventional medical treatment for cognitive decline. However, as a simple, convenient, inexpensive, and effective traditional Chinese medical method, acupuncture is widely recognized in medical science for its effectiveness in treating various diseases, particularly a needling technique for restoring cognition, which has been widely used for patients with VCI, giving hope for the treatment of VCI [29,30].

Chinese medicine classifies VCI as “forgetfulness” and “dullness” and considers the condition to be located in the brain. The governor meridian oversees all of the yang and has the ability to regulate both yin and yang, replenish qi and blood, fill the marrow, awaken the mind, and open the orifices. Therefore, the governor meridian is preferred for the treatment of brain diseases [31,32]. Our group’s previous clinical research revealed that electroacupuncture head acupuncture points can effectively improve cognitive dysfunction after stroke, improve patients’ ability to take care of themselves in daily life, and improve the rehabilitation of patients’ limb motor function [14,33]. More importantly, we are now committed to studying the possible mechanisms of TCM in treating diseases from the perspective of modern medicine.

Iron is essential for many biological processes in the central nervous system, including oxygen transport, myelin production, and neurotransmitter synthesis and metabolism [34]. A number of studies have suggested that disorders of iron metabolism may be an important pathophysiological factor in VCI [35-38]. Clinical studies have reported not only a significant increase in serum iron ions in patients with a history of stroke but also a significant increase in iron deposition in brain tissue, as observed using imaging methods [28,39]. Animal experiments have revealed the presence of iron accumulation in the affected brain regions of experimental animals with cerebral ischemia or reperfusion injury [40]. Another clinical study found brain iron accumulation in patients with subcortical ischemic VD and its correlation with the severity of cognitive impairment [41]. Therefore, regulation of brain iron deposition and somatic iron metabolism is expected to provide a new target for the treatment of VCI. Based on this, we propose a randomized controlled trial to verify whether the improvement of cognitive function in patients with VD by Tongdu Xingshen acupuncture is associated with the modulation of cerebral iron deposition and body iron metabolism.

### Innovations and Limitations

This is the first clinical randomized experimental protocol of our team to use iron metabolism as an entry point to explore the potential mechanisms of acupuncture for VCI using modern medicine. This trial will be conducted using rigorous methods, for example, the patients with VCI will be randomly assigned to 2 groups; the data will undergo blind statistical analysis; and the interventionists, efficacy evaluators, and statisticians will be separated. However, due to the specificity of acupuncture, this experimental protocol could not be double blinded. Moreover, we could not avoid gender bias during the random allocation process. Therefore, to ensure the reliability of the data, we still need to further expand the sample size.

### Conclusions

This study will provide data on the effects of Tongdu Xingshen acupuncture on cerebral iron deposition as well as somatic iron metabolism in patients with VCI. More importantly, if the hypothesis of this study is confirmed, the results would provide evidence supporting TCM acupuncture as an alternative treatment modality for VCI and lay the scientific basis for the clinical application of Tongdu Xingshen acupuncture for VCI, which may have important socioeconomic benefits.

## Abbreviations

MMSE: Mini-Mental State Examination
MoCA: Montreal Cognitive Assessment
QSM: quantitative susceptibility mapping
SWI: sensitivity-weighted imaging
TCM: traditional Chinese medicine
VCI: vascular cognitive impairment
VD: vascular dementia

## Data Availability

The data sets generated and analyzed during this study are available from the corresponding author on reasonable request.
